# New Treatment Strategies for IgA Nephropathy: Targeting Plasma Cells as the Main Source of Pathogenic Antibodies

**DOI:** 10.3390/jcm11102810

**Published:** 2022-05-16

**Authors:** Dita Maixnerova, Delphine El Mehdi, Dana V. Rizk, Hong Zhang, Vladimir Tesar

**Affiliations:** 1 Department of Nephrology, First Faculty of Medicine, General University Hospital, Charles University, 128 08 Prague, Czech Republic; vladimir.tesar@lf1.cuni.cz; 2 MorphoSys, MorphoSys US Inc., Boston, MA 02210, USA; delphine.elmehdi@morphosys.com; 3 Division of Nephrology, University of Alabama at Birmingham, Birmingham, AL 35294, USA; drizk@uabmc.edu; 4 Renal Division, Peking University First Hospital, Peking University Institute of Nephrology, Beijing 100034, China; hongzh@bjmu.edu.cn

**Keywords:** IgA nephropathy, galactose-deficient IgA1, plasma cells, CD38, renal pathology

## Abstract

Immunoglobulin A nephropathy (IgAN) is a rare autoimmune disorder and the leading cause of biopsy-reported glomerulonephritis (GN) worldwide. Disease progression is driven by the formation and deposition of immune complexes composed of galactose-deficient IgA1 (Gd-IgA1) and Gd-IgA1 autoantibodies (anti-Gd-IgA1 antibodies) in the glomeruli, where they trigger complement-mediated inflammation that can result in loss of kidney function and end-stage kidney disease (ESKD). With the risk of progression and limited treatment options, there is an unmet need for therapies that address the formation of pathogenic Gd-IgA1 antibody and anti-Gd-IgA1 antibody-containing immune complexes. New therapeutic approaches target immunological aspects of IgAN, including complement-mediated inflammation and pathogenic antibody production by inhibiting activation or promoting depletion of B cells and CD38-positive plasma cells. This article will review therapies, both approved and in development, that support the depletion of Gd-IgA1-producing cells in IgAN and have the potential to modify the course of this disease. Ultimately, we propose here a novel therapeutic approach by depleting CD38-positive plasma cells, as the source of the autoimmunity, to treat patients with IgAN.

## 1. Introduction

Immunoglobulin A nephropathy (IgAN), a rare autoimmune disorder, is the leading cause of biopsy-reported glomerulonephritis (GN) worldwide [1,2]. The global incidence of IgAN is 2.5 per 100,000, and approximately 40% of patients progress to end-stage kidney disease (ESKD) within 20 years of diagnosis [3,4]. Upon the onset of ESKD, lifelong dialysis or kidney transplantation is required and substantially increases disease burden [2]. Dialysis can decrease both physical and mental quality of life due to fatigue and decreased ability to work, which correspond with expected social consequences and increased economic burden [5,6,7]. Survival rates after starting dialysis for IgAN patients are favorable (93% and 65% at 10 and 20 years, respectively) compared with those with other glomerulonephritis (64% and 32% at 10 and 20 years, respectively). This survival advantage is mostly related to the younger age of IgAN patients on dialysis; however, dialysis can increase the risk of infection and cardiovascular complications [2,8,9]. Progression to ESKD also increases mortality risk and can result in a six-year reduction in median life expectancy [10]. Transplantation occurs more frequently in people with IgAN compared with other ESKDs as patients are typically younger and have fewer comorbidities [11]. Transplantation can decrease mortality and improve quality of life, but is also associated with iatrogenic infection, disease recurrence, and risk of transplant failure [2,12,13].

**Figure 1:**
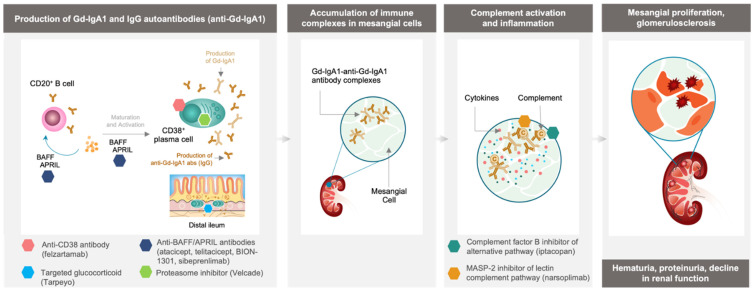
Immunopathogenesis of IgAN and potential therapeutic targets. Early CD20^+^ B cells produce small amounts of antibodies (Gd-IgA1), whereas CD38^+^ plasma cells can produce both these antibodies and high quantities of autoantibodies (anti-Gd-IgA1). The antibodies and autoantibodies form Gd-IgA1 and anti-Gd-IgA1 antibody immune complexes that can deposit and accumulate in mesangial cells. The deposition of immune complexes activates the alternative and lectin pathways of the complement system leading to chronic inflammation, which contributes to podocyte damage and, ultimately, loss of renal function that manifests in patients as hypertension, hematuria, proteinuria and reduction in glomerular filtration rate. The targets of novel therapies aim to inhibit pathogenesis by affecting the immune system at various stages of pathogenesis. Atacicept and telitacicept are recombinant fusion proteins able to bind cytokines BAFF and APRIL and interfere with B cell and plasma cell survival. BION-1301 and sibeprenlimab are monoclonal antibodies targeting the cytokine APRIL, which may reduce levels of Gd-IgA1 and IgG autoantibodies. Velcade^®^ is a proteasome inhibitor that targets and depletes plasma cells. Felzartamab, an anti-CD38 antibody, is designed to target the highly expressed CD38 cell surface antigen on plasma cells. Tarpeyo™, a targeted-release glucocorticoid that aims at the highest concentration of Peyer’s patches in the distal ileum to reduce production of Gd-IgA1. Iptacopan, a small molecule factor B inhibitor of the alternative complement pathway, acts to reduce damage caused by accumulation of immune complexes in the mesangial cells. Narsoplimab, a MASP-2 monoclonal antibody, acts as an inhibitor of the lectin complement pathway. Abs, antibodies; APRIL, a proliferation inducing ligand; BAFF, B cell activating factor; CD, cluster of differentiation; GD-IgA1, galactose-deficient immunoglobulin A1; GFR, glomerular filtration rate; MASP, mannan-binding lectin serine protease.

Data suggest that genetic and environmental factors play a role in the pathogenesis of IgAN by triggering production of galactose-deficient IgA1 (Gd-IgA1) and, subsequently, Gd-IgA1 autoantibodies (anti-Gd-IgA1 antibodies) [1,14]. Both Gd-IgA1 and anti-Gd-IgA1 antibody production are believed to be driven by CD38^+^ plasma cells, a cell type that contributes to autoimmune disorders by producing high quantities of autoantibodies [15,16,17]. Together, Gd-IgA1 and anti-Gd-IgA1 antibodies form immune complexes that accumulate in the glomerular mesangium and induce activation of the complement system leading to chronic inflammation, mesangial proliferation, glomerulosclerosis, and loss of renal function (Figure 1) [1,18,19,20]. Levels of Gd-IgA1, anti-Gd-IgA1 antibodies, and the resulting immune complexes are biomarkers for disease severity and progression in patients with IgAN. Estimated glomerular filtration rate (eGFR) decline and poor renal survival are associated with higher Gd-IgA1 and anti-GdIgA1 antibody levels [17,21]. 

A differential diagnosis for IgAN is important as other conditions can present similarly but pathological mechanisms of disease and care management differ. IgA-dominant infection-associated glomerulonephritis can be challenging to distinguish from primary IgAN but imperative as immunosuppressive therapy; a standard for the latter would be contraindicated for the former. In addition, IgA vasculitis with nephritis (IgAV), previously called Henoch-Schönlein Purpura, has historically been thought of as a systemic disease on the same spectrum as IgAN. Although the two entities share some mutual steps in the pathogenesis, the signature presence of skin rash, extra-renal symptoms, and earlier age at onset indicate IgAV may require different treatment approaches and hence has been excluded from all ongoing clinical trials.

Historically, treatment of IgAN has been focused on supportive treatments that correct hypertension and proteinuria and may slow down the loss of eGFR in some patients, but most patients presenting with higher residual proteinuria still progress to ESKD. Selecting supportive care is often contingent upon balancing risks and benefits for each patient with the goal of preventing ESKD [22,23,24]. Renin-angiotensin system (RAS) inhibitors are often utilized to manage hypertension and slow down decreases in eGFR. There are limited risks with use of RAS inhibitors but their efficacy to prevent ESKD in high-risk patient is also limited [24,25]. An investigational dual inhibitor of the angiotensin II type 1 (AT1) and endothelin type A (ET-A) receptors, sparsentan (Travere Therapeutics, San Diego, CA, USA), may provide improved protection and reduce proteinuria levels to a greater extent than current supportive care regimens [24,26,27]. Sodium-glucose cotransporter-2 (SGLT2) inhibitor, dapagliflozin (Farxiga, AstraZeneca, Cambridge, UK) has also shown promise as a supportive treatment to decrease proteinuria and ameliorate the progression of chronic kidney disease [28,29]. Systemic corticosteroids are used to treat intermediate and high-risk patients with IgAN; however, some studies showed their limited efficacy and potentially severe toxicity [30,31].

With the risk of progression and limited treatment options, there is an unmet need for therapies that address the key mechanism of disease, which is the formation of pathogenic Gd-IgA1 containing immune complexes. In 2020, a comprehensive review was published that described therapeutic candidates that target immune components thought to contribute to disease progression [24]. Since that time, the U.S. Food and Drug Administration (FDA) granted accelerated approval for TARPEYO™ (targeted-release budesonide formulation, Calliditas Therapeutics, Stockholm, Sweden), as the first disease-specific treatment for IgAN and strengthened an ongoing discussion around targeting the primary cause of the disease, Gd-IgA1-producing cells, which are upstream of immune complex formation [32,33].

This article will review therapies, both approved and in development, that support the depletion of Gd-IgA1 and anti-Gd-IgA1 antibody-producing cells in IgAN and have the potential to modify the course of this disease (Table 1). 

B cells and plasma cells both play a role in immunologic memory and play a role in autoimmune disease but regulation, location, and cell surface expression differs between these two cell types [34,35]. Plasma cells, in particular long-lived plasma cells, are capable of secreting antibodies for several years or even a lifetime. Due to their longevity, long-lived plasma cells play a crucial role in protective immunity and autoimmunity [16,34]. Plasma cells are known to be major producers of antibodies due to their expanded endoplasmic reticulum, and may represent a primary source of Gd-IgA1 and anti-Gd-IgA1 antibodies, which underlie initiation and progression of IgAN [1,16]. An increasing number of studies have revealed that Gd-IgA1 could be derived from primed plasma cells in the mucosa [36,37,38,39]. The role of plasma cells in IgAN is supported by data that show patients have significantly higher percentages of CD38^+^ cells than healthy controls [40]. Upregulation of Toll-like receptor 9 (TLR9), higher serum levels of B cell activating factor (BAFF) and increased expression of a proliferation-inducing ligand (APRIL) have been linked to proliferation, activation, and long-term maintenance of antibody and autoantibody producing plasma cells in IgAN [41,42]. Unlike B cells, plasma cells are characterized by a high cell surface expression of CD38 and a loss of CD20 [16,34]. This is likely why anti-CD20 antibody therapeutics, such as rituximab, are able to deplete B cells but fail to eliminate plasma cells or reduce serum levels of Gd-IgA1 or anti-Gd-IgA1 antibodies [24,34,35,43,44]. A randomized controlled trial evaluating rituximab in IgAN showed no clinical benefit compared with standard therapy [44].

We propose here to explore data from approved therapies and compounds in development, as well as preliminary data on anti-CD38 antibody therapy, felzartamab, that support specific targeting of plasma cells.

## 2. New Strategies for the Management of IgAN

The immune system plays a multifaceted role in initiating and promoting the loss of kidney function seen in patients with IgAN [1,16,17,20]. Growing clinical data support approaches that deplete Gd-IgA1-producing cells or reduce immune complex-mediated inflammation, which hold greater promise than broad-acting immunosuppressive treatments (ISTs) that have historically demonstrated a lack of efficacy or were associated with significant toxicity.

## 3. Inhibition of Immune Complex-Activated Complement Activity

There is pathologic biochemical and genetic data supporting the pivotal role of the complement system in the pathogenesis and progression of IgAN that is now well established [20]. Accumulating evidence suggests that activation of both the alternative and lectin pathways, leads to glomerular inflammation and injury in IgAN [20,43,45,46,47]. Here, we review several complement inhibitors that are in advanced stages of clinical development.

Iptacopan (LNP023, Novartis, Basel, Switzerland) is an investigational, oral small molecule complement factor B inhibitor of the alternative pathway that is being evaluated in adults with IgAN [48,49]. Results from a Phase 2 clinical trial (NCT03373461) demonstrated a potential for effective and clinically meaningful reduction in proteinuria. The trial randomized 112 patients with IgAN into three dosing arms of iptacopan and a placebo arm. Results showed the highest dose of iptacopan (200 mg, twice daily) can reduce urine protein: creatinine ratio (UPCR) by 40% from baseline to 6 months, compared with placebo. Based on these encouraging data, the Phase 3 APPLAUSE-IgAN trial has been launched and is currently ongoing (NCT04578834) [48,50].

Similarly, narsoplimab (OMS721, Omeros, Seattle, WA, USA), an investigational humanized monoclonal antibody selectively targeting mannan-binding lectin-associated serine protease-2 (MASP-2), is a novel pro-inflammatory protein target and the effector enzyme of the lectin pathway that is being evaluated in patients with IgAN [20,51,52]. Three-year follow-up data from a Phase 2 clinical trial (NCT02682407) in 12 high-risk patients with advanced IgA nephropathy showed a median reduction in proteinuria of 64.4% and long-term improvement or sustained stabilization in eGFR when treated with narsoplimab [51,53]. A Phase 3 trial is currently ongoing (NCT03608033) [20].

Iptacopan and narsoplimab target the alternative and lectin pathways, respectively, leaving the classical complement pathway intact and able to respond to pathogens [54,55].

While complement inhibition has the potential to reduce proteinuria and slowing down eGFR loss, continued production and deposition of immune complexes in the glomeruli may require long-term treatment with complement inhibitors to prevent the progression of kidney disease. 

## 4. Depletion of Gd-IgA1-Producing Immune Cells

New treatment strategies aim to reduce immune complex formation and subsequent inflammation by targeting sources of Gd-IgA1 and its anti-Gd-IgA1 antibody production.

### 4.1. Targeting Cytokines Responsible for B Cell and Plasma Cell Activation and Survival

Under normal conditions, B cells and plasma cells play an important role in producing antibodies that help defend against a multitude of infections. In autoimmune disorders, these same cells can exacerbate or contribute to the disease by producing autoantibodies [15,56]. BAFF and APRIL, cytokines from the tumor necrosis factor family, are known to mediate B cell and plasma cell function and survival [57]. BAFF and APRIL can activate the NF-kB pathway by binding to several cell surface receptors, including transmembrane activator and calcium-modulator and cyclophilin ligand interactor (TACI), which promotes plasma cell survival and can stimulate IgG antibody production [57,58]. Belimumab (Benlysta, GSK, Brentford, UK), a monoclonal antibody targeting soluble BAFF, is designed to inhibit activation of B cells and plasma cells thought to drive production of pathogenic antibodies in several autoimmune disorders [59]. Belimumab has been approved for systemic lupus erythematosus (SLE) and/or lupus nephritis, and is currently being investigated in addition to rituximab in a Phase 2 trial (NCT03949855) for primary membranous nephropathy (MN), an autoimmune kidney disease with up to 20% chance of progression to ESKD [60,61]. 

Increased APRIL expression has been observed in patients with IgAN and is correlated with increased expression of Gd-IgA1 antibodies [58]. Targeting APRIL has the potential to limit antibody production in autoimmune-associated plasma cells. This concept is supported by results from the first cohort of a Phase 1/2 study (NCT03945318) evaluating BION-1301 (Chinook Therapeutics, Seattle, WA, USA), an anti-APRIL monoclonal antibody, in up to 40 patients with IgAN showing sustained reduction in levels of Gd-IgA1 antibodies and proteinuria [62]. Similarly, dual targeting of both BAFF and APRIL with atacicept (Vera Therapeutics, Brisbane, CA, USA), a soluble TACI-Ig fusion protein, showed reduction in Gd-IgA1 antibody levels and proteinuria when evaluated in a Phase 2 study (NCT02808429) in 16 patients with IgAN [63]. Atacicept is being evaluated for IgAN in a Phase 2b trial (ORIGIN; NCT04716231) [64]. In addition, a Phase 2 trial with telitacicept (RemeGen, Yantai, China), a soluble TACI-Ig fusion protein, in 44 patients with IgAN also showed the proteinuria reduction (NCT04905212) [65,66]. Anti-APRIL antibody, sibeprenlimab (VIS649, Visterra/Otsuka, Cambridge, MA, USA/Tokyo, Japan), is being studied for safety and efficacy in a Phase 2 clinical trial (NCT04287985) [67,68]. 

These data support BAFF and APRIL inhibition as a potential treatment for IgAN; however, further studies are required to understand the broader impacts on immunogenicity when altering function of both B cells and plasma cells.

### 4.2. Tarpeyo, a Targeted Approach for Immune Cell Depletion in the Small Intestine

Gut-associated lymphoid tissue (GALT), including Peyer’s Patches, which are thought to contain a high concentration of conventional surface IgA1-expressing primed mucosal B cells and plasma cells, may be responsible for the production of Gd-IgA1 in IgAN [69,70]. Although other studies suggest the nasopharynx-associated lymphoid tissue (NALT) or palatine tonsils may also play an important role in Gd-IgA1 production in patients with IgAN [71,72]. Undoubtedly, reducing Gd-IgA1 levels is a promising approach for disease modification [36]. Targeted corticosteroids, such as targeted-release budesonide (Tarpeyo), have been shown to provide effective modulation and reduction in the immune cells within the gut, including long-lived plasma cells and memory B cells [32,69,73]. Tarpeyo is designed to deliver a delayed release formulation of budesonide to the distal ileum, where it locally suppresses immune cell activity, including Gd-IgA1-producing cells and reduce circulating immune complexes that cause downstream inflammation and kidney impairment [69,74,75]. 

In a Phase 2b study including 150 patients with IgAN and persistent proteinuria despite optimized RAS blockade (NEFIGAN; NCT01738035), Tarpeyo significantly reduced proteinuria levels and stabilized kidney function [76]. Subsequent results from the treatment period of a Phase 3 study (NefIgArd; NCT03643965) evaluating Tarpeyo in 360 patients with IgAN and persistent proteinuria despite optimized RAS blockade showed treatment with Tarpeyo reduced proteinuria by 27% and stabilized eGFR at 9 months compared with placebo, which led to its conditional accelerated approval by the FDA [77,78]. 

These data suggest that reducing activity of Gd-IgA1-producing immune cells, which would also reduce levels of anti-Gd-IgA1 antibodies and subsequent immune complex formation and deposition, could improve patient outcomes. However, further evidence is needed to confirm that Tarpeyo reduces Gd-IgA1-producing immune cells in Peyer’s Patches.

### 4.3. Velcade, Plasma Cell Depletion via Proteasome Inhibition

Additional support for depletion of plasma cells has been observed with VELCADE^®^ (bortezomib, Millennium/Takeda, Cambridge, MA, USA), a proteasome inhibitor that depletes plasma cells and is approved for treatment of MM, has also shown promise in an open-label pilot trial (NCT01103778) which enrolled 8 people with IgAN. Results showed three participants achieved complete remission (proteinuria of <300 mg/day) after treatment for 1 year, suggesting targeting plasma cells through proteasome inhibition could reduce proteinuria in patients with IgAN [79]. However, larger trials are needed to better assess safety and efficacy in patients with IgAN. 

### 4.4. Felzartamab, Targeted CD38^+^ Plasma Cell Depletion

The clinical data supporting targeting of Gd-IgA1-producing immune cells in the gut showed improved outcomes for patients but not complete resolution of the disease [77]. Targeting multiple locations and types of autoantibody-producing plasma cells and thereby potentially reducing Gd-IgA1 as well as anti-Gd-IgA1 antibody levels at the same time may contribute to a more robust improvement in patient outcomes. 

**Figure 2:**
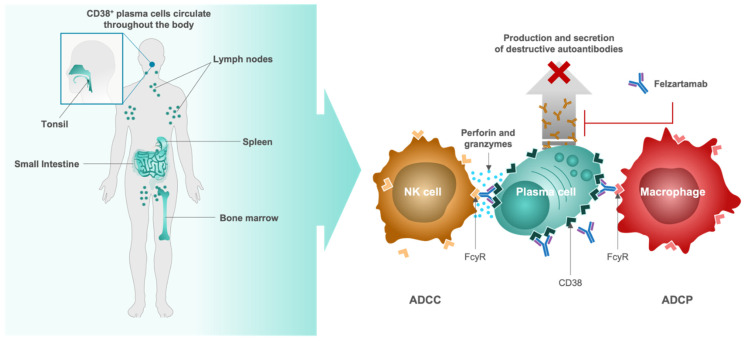
Proposed mechanism of action of felzartamab (MOR202/TJ202) for depleting antibody and auto-antibody-producing CD38^+^ plasma cells. ADCC, antibody-dependent cell-mediated cytotoxicity; ADCP, antibody-dependent cell mediated phagocytosis; CD, cluster of differentiation; FcγR, Fc-gamma receptor; NK, natural killer.

Felzartamab (MOR202/TJ202, MorphoSys, Planegg, Germany), a fully human immunoglobulin G1 (IgG1) monoclonal antibody designed to target the highly expressed CD38 cell surface antigen on plasma cells, is being evaluated as a potential first-in-class immunotherapy in a Phase 2a clinical trial for patients with IgAN (IGNAZ; NCT05065970) [80]. Binding of felzartamab to CD38^+^/CD20^−^ plasma cells is thought to induce cell killing through two complementary mechanisms of action (MoA) including antibody-dependent cell-mediated cytotoxicity (ADCC) via natural killer cells and antibody-dependent cell-mediated phagocytosis (ADCP) via macrophages (Figure 2) [16,81,82,83]. Complement-dependent cytotoxicity (CDC) is described to play a role in infusion-related reactions (IRRs), but based on in vitro testing, felzartamab does not trigger CDC or anti-drug antibodies [82,84]. 

Preliminary results from a Phase 1b/2a, proof-of-concept trial (M-PLACE, NCT04145440) of felzartamab in 31 patients with anti-phospholipase A2 receptor (PLA2R) antibody-positive MN showed a 46.1% reduction in pathogenic anti-PLA2R autoantibody levels after 1 week in 89% (24/27) of patients with evaluable results. The reduction was sustained, and most patients showed a further increase in reduction over time (12-week treatment). These results support successful and sustained depletion of CD38^+^ plasma cells with felzartamab [85]. Although further trials are required to collect safety data and address any concerns about therapies that modulate the immune system, the safety profile of felzartamab in the M-PLACE trial was found to be consistent with the proposed MoA, and treatment-emergent adverse events were manageable in patients with MN [85]. Treatment-emergent adverse events (TEAEs) occurred in 26/31 patients and were mostly mild or moderate in severity and the majority resolved. A total of 5 patients experienced treatment-emergent serious adverse events, 2 of which were related to felzartamab (type-I hypersensitivity and grade 3 IRR), and no events resulted in death [86]. 

Anti-CD38 activity has also been established for felzartamab in multiple myeloma (MM) clinical trials. In a Phase 1/2a clinical trial (NCT01421186) evaluating felzartamab in 91 adults with relapsed/refractory (r/r) MM, felzartamab reduced M-protein levels, supporting systemic depletion of CD38^+^ plasma cells [82,87]. Limited downregulation of CD38 on MM cells was also observed in patients treated with felzartamab, indicating potential for sustained efficacy [88]. 

Taken together, the preliminary efficacy and safety data on felzartamab in MN and the proof-of-concept results in r/r MM provide further support for clinical development of anti-CD38 antibody therapies in IgAN and highlight their potential application in other plasma cell-driven autoimmune diseases. 

As with any immunomodulatory therapies, there is an increased risk of infection due to downregulation of the natural immune defenses. With therapeutics such as complement inhibitors, there is a potential for disrupting the innate immune system, which is one of the first immunologic responses to pathogenic bacteria [89]. The complement pathway, however, does have redundancies within the three main pathways—classical, lectin and alternative—that allow for therapeutic targeting of this pathway while mitigating risk of infection [54,55]. When depleting B cells with general immunosuppression therapies, such as systemic glucocorticoids, there is potential to disrupt the adaptive immune system, which is responsible for clearing bacteria, viruses, fungi, and parasites [90]. Taking a more targeted approach, as is the case with several of the therapies in development, could reduce the risk of infection and potentially improve the risk/benefit profile in patients treated for IgAN. 

## 5. Conclusions

IgAN is an autoimmune disease with a disease burden that greatly disrupts patients’ lives and increases their risk for chronic kidney disease and, ultimately, ESKD. Safe and effective disease-modifying agents that target the source of the disease are greatly needed for this patient population. As the scientific community has learned more about the immunopathology of this disease, new approaches are being investigated that may slow or stop disease progression by targeting the underlying disease triggers.

Preliminary data evaluating complement inhibitors in IgAN, which act downstream of plasma cells and immune complex formation, indicate their potential to improve kidney function by reducing levels of chronic inflammation that contribute to disease progression. This approach, however, is aimed at preventing or ameliorating damage but cannot suppress the ongoing production of pathogenic autoantibodies.

Targeting B cell and plasma cell activators BAFF and APRIL have emerged as a promising approach for reducing Gd-IgA1 antibody and autoantibody production. Early clinical studies evaluating both BION-1301, atacicept, and telitacicept have shown that targeting APRIL and BAFF may reduce antibody levels and proteinuria. 

An alternative approach is to target the tissues and cell types most associated with production of disease-causing antibodies (Gd-IgA1). This approach was recently validated through approval of targeted release budesonide (Tarpeyo). The clinical data on Tarpeyo showed substantial benefit for patients with IgAN over current standard of care, indicating the potential of a therapy that can interrupt immunopathogenesis by reducing mucosal production of Gd-IgA1. Tarpeyo is designed to act locally on immune cells in the small intestine, potentially missing other sources of Gd-IgA1-producing cells throughout the body, including NALT and tonsils. As plasma cells are found in mucosa throughout the body, specifically targeting all plasma cells may provide even more robust reduction in circulating pathogenic antibodies and immune complexes by removing the main source of autoantigen and/or autoantibody production in all lymphoid tissues [36,37,38,39]. Furthermore, targeting CD38 selectively targets plasma cells and allows for continued immune protection by CD38 negative B cells. The anti-CD38 antibody therapy, felzartamab, has shown successful depletion of plasma cells in the autoimmune-driven nephropathy MN and hematologic cancers, such as MM. An anti-CD38 approach may also have great potential for treatment of patients with IgAN. 

Altogether, these data support development of strategies that deplete plasma cells, which aim to stop generation of Gd-IgA1 and anti-Gd-IgA1 antibodies and potentially reduce immune complex deposition, inflammation, and tissue damage, thereby preserving kidney function in patients with IgAN. However, modulating the immune system comes with an increased risk of infection and more clinical data is needed to understand the long-term effects of treatment with these novel immunotherapies.

## Figures and Tables

**Table 1:** Therapies in Clinical Development for Treatment of IgAN.

Agent	Target	Modality	Mechanism of Action
**Atacicept**	BAFF and APRIL	Fusion protein/antibody	Inhibits maturation and activation of B cells
**BION-1301**	APRIL	Monoclonal antibody	Inhibits maturation and activation of B cells
**Felzartamab (MOR202/TJ202)**	CD38	Monoclonal antibody	Depletes CD38^+^ plasma cells
**Iptacopan**	Factor B	small molecule	Inhibits complement alternative pathway activation
**Narsoplimab**	MASP-2	Monoclonal antibody	Inhibits complement lectin pathway activation
**Sibeprenlimab**	APRIL	Monoclonal antibody	Inhibits maturation and activation of B cells
**Tarpeyo (targeted-release budesonide)**	Glucocorticoid receptors	Corticosteroid	Depletes B cells and plasma cells in the small intestine
**Telitacicept**	BAFF and APRIL	Fusion protein/antibody	Inhibits maturation and activation of B cells
**Velcade (bortezomib)**	Proteasome	Peptide	Inhibits proteasome activity in plasma cells
